# Apparent diet digestibility of captive colobines in relation to stomach types with special reference to fibre digestion

**DOI:** 10.1371/journal.pone.0256548

**Published:** 2021-09-20

**Authors:** Satoru Hoshino, Satoru Seino, Takashi Funahashi, Tomonori Hoshino, Marcus Clauss, Ikki Matsuda, Masato Yayota

**Affiliations:** 1 The United Graduate School of Agricultural Science, Gifu University, Gifu, Japan; 2 Zoo Biology Research Center, Gifu University, Gifu, Japan; 3 Preservation and Research Center, City of Yokohama, Yokohama, Japan; 4 Japan Monkey Centre, Inuyama, Aichi, Japan; 5 Vetsuisse Faculty, Clinic for Zoo Animals, Exotic Pets and Wildlife, University of Zürich, Zürich, Switzerland; 6 Chubu University Academy of Emerging Sciences, Kasugai-shi, Aichi, Japan; 7 Wildlife Research Center of Kyoto University, Sakyo, Kyoto, Japan; 8 Institute for Tropical Biology and Conservation, Universiti Malaysia Sabah, Kota Kinabalu, Sabah, Malaysia; 9 Faculty of Applied Biological Sciences, Gifu University, Gifu, Japan; Universidad de Guadalajara, MEXICO

## Abstract

Colobine monkeys are known for the anatomical complexity of their stomachs, making them distinct within the primate order. Amongst foregut fermenters, they appear peculiar because of the occurrence of two different stomach types, having either three (‘tripartite’) or four (‘quadripartite’, adding the praesaccus) chambers. The functional differences between tri and quadripartite stomachs largely remain to be explained. In this study, we aim to compare the apparent digestibility (aD) in tripartite and quadripartite colobines. Hence, we measured the aD in two colobine species, *Nasalis larvatus* (quadripartite) and *Trachypithecus cristatus* (tripartite), in two zoos. We also included existing colobine literature data on the aD and analysed whether the aD of fibre components is different between the stomach types to test the hypothesis of whether quadripartite colobines show higher aD of fibre components than tripartite colobines did. Our captive *N*. *larvatus* specimen had a more distinctively varying nutrient intake across seasons with a larger seasonal variation in aD than that of a pair of *T*. *cristatus*, which mostly consumed commercial foods with a lower proportion of browse and less seasonal variation. We observed higher aD of dry matter (DM), neutral detergent fibre (NDF) and acid detergent fibre (ADF) in the *N*. *larvatus* specimen, suggesting a higher gut capacity of *N*. *larvatus* provided by the additional praesaccus forestomach chamber. Based on the analysis of literature data for aD, we also found that quadripartite species achieved higher fibre digestibility at similar dietary fibre levels compared with tripartite species, supporting the hypothesis that the additional gut capacity offered by the praesaccus facilitates a longer retention and hence more thorough microbial fermentation of plant fibre.

## Introduction

Primates display a diverse array of digestive adaptations while covering various trophic niches, from folivory to frugivory, gummivory, insectivory, omnivory and nearly exclusive carnivory in some human populations [1,2]. In particular, Old World monkeys of the subfamily Colobinae are known for the anatomical complexity of their stomachs, making them distinct within the primate order as the only ‘foregut fermenters’. Their stomachs are complex and multi-chambered, harbouring a symbiotic microbiome that digest plant fibre and detoxify defensive plant chemicals, possibly allowing them to exploit a diet of leaves in greater quantities than other simple-stomached primates [3,4]. Because of such anatomical complexity with their folivory, colobines have historically often been considered difficult to maintain healthy under zoo feeding regimens, especially when compared with frugivorous and/or omnivorous primates with simple stomachs [5,6].

In previous studies, two different types of colobine forestomach have been distinguished. The so-called ‘tripartite’ type comprises a saccus, tubiform and glandular stomach part; these can be found in all colobines. The ‘quadripartite’ type has an additional blind sac, or pouch, named ‘praesaccus’, which is thought to represent an additional chamber. It is found in the genera *Procolobus*, *Piliocolobus*, *Rhinopithecus*, *Pygathrix* and *Nasalis* [7–9]. Notably, genera with a quadripartite stomach are notoriously difficult to maintain and breed in captivity, especially in temperate regions [10–13] where constant supply of fresh browse is especially difficult in autumn and winter, and chemical composition of fresh browse differs across plant species and seasons [14]. Therefore, to reduce gastrointestinal disorders and enhance health and survival in captive colobines, identifying an appropriate diet considering nutritional differences across plant species and seasons, in relation to their digestive physiology, is one of the goals for *ex situ* animal management.

The functional differences between tri and quadripartite stomachs, however, largely remain unexplained. Matsuda, Chapman and Clauss [15] compiled literature data on the natural diet of colobine species to investigate the role of the praesaccus, suggesting that a larger gut capacity provided by an additional praesaccus is an important characteristic by which colobines survive on diets with a particularly high proportion of leaves. Thus, the higher intake capacity for species with quadripartite stomach would be assumed to be detrimental in the case of more digestible (commercial) diets in captivity than those in the wild, thereby leading to malfermentation by highly digestible components such as sugars or starch [5,6]. Conversely, it may be assumed that species with tripartite stomachs are less susceptible to extreme bouts of malfermentation when fed highly digestible diets, simply because of their relatively reduced intake capacity. Evidently, these speculations remain to be tested.

One approach is to compare the apparent digestibility (aD), i.e. the ratio of the difference of the ingested and faecal nutrients to the ingested nutrients, in tripartite and quadripartite colobines to obtain information relevant for evaluating the digestive capacity for fibre. The aD evaluates the ability to break down and absorb nutrients, like fibre contained in browse/leaves, and thus measuring aD facilitates insights into the digestive adaptations and capacities of a species, and–maybe more importantly–also comparisons between species or species groups. It has previously been quantified in some colobines in comparison to simple-stomached primates, indicating that colobines show higher aD of fibre components, e.g. neutral detergent fibre (NDF) and acid detergent fibre (ADF), than simple-stomached primates, such as *Macaca fuscata* [16], *Alouatta* spp. [17] and *Nomascus siki* [18]. However, to our knowledge, a study focusing on comparing aD between tripartite and quadripartite colobines has not yet been undertaken. In our comparison of aD between the colobines with different stomach types, we expected that quadripartite species with a putatively larger gut capacity would display higher aD of fibre than tripartite ones.

As a first preliminary approach to this question, we examined the aD of two captive colobine species, *Nasalis larvatus* (quadripartite) and *Trachypithecus cristatus* (tripartite), in two temperate region zoos. We focused on the seasonal difference of their aD to evaluate the effects of seasonal variation in nutrient composition throughout the year on their digestive efficiency. Additionally, to test the hypothesis of whether quadripartite colobines show higher aD of fibre components than tripartite colobines, we included existing colobine literature data on the aD and analysed whether the aD of fibre components is different between the stomach types.

## Materials and methods

### Ethics statement

We conducted the feeding experiments of proboscis monkey (*N*. *larvatus*) and silvered langur (*T*. *cristatus*) in the Yokohama Zoological Gardens, Zoorasia (approval ID: #256) and Japan Monkey Centre in Japan (approval ID: #2018–016), respectively. Invasive or stressful approaches such as capture, manual restraint or anaesthesia were not performed in this study. The materials were collected from animals non-invasively. This study was approved by the Welfare of Gifu University (approval ID: #17092). All animal experiment procedures were conducted following the Guidelines for Proper Conduct of Animal Experiments (Science Council of Japan, 2006; http://www.scj.go.jp/ja/info/kohyo/pdf/kohyo-20-k16-2e.pdf) and the Guidelines of Animal Research and Welfare of Gifu University (2008; https://www.gifu-u.ac.jp/20150821-12a-experi.pdf).

### Digestion trials

In the Yokohama Zoological Gardens, the experiments were performed with one male *N*. *larvatus* (14 years old) housed individually. Three digestive trials were conducted in different seasons: autumn (3rd–16th September 2017), winter (2nd–15th January 2018) and summer (8th–15th June 2018). Each trial was composed of two different continuous periods: the acclimatisation (seven days) and sampling (seven days) periods. We were compelled to shorten one acclimatisation (8–12 June 2018) and sampling (13–15 June 2018) period due to heavy rain that soaked animal faeces and leftover leaves in the cage. Note that since the mean retention time for different markers in the whole digestive tract of *N*. *larvatus* has been reported as approximately 40 h [19], we believe that the shorter sampling period (72 h), though not ideal, is still suitable for assessing digestibility in this species. The animal was fed a mixed diet of seasonally available fresh leaves with branches and twigs harvested nearby the zoo and supplied by several commercial company or private farms (Ogawana farm, Dairi-en and Shiramori-en, Yokohama and Ohnishi Agricultural Corp. Yagazishima farm, Nago), vegetables and commercial pellets (S1 Table) two times daily. Water was freely available at all times.

In the Japan Monkey Centre, the trials were performed with two adult male *T*. *cristatus* (16 and 17 years old) housed together. Two experiments were conducted in different seasons: summer (30 July to 12 August 2018) and winter (14–27 February 2019) applying the same design as in *N*. *larvatus*, i.e. two continuous periods. Note that as their diets commonly fed did not differ in composition as much throughout the year compared to those in *N*. *larvatus*, we assessed the diets and aD of *T*. *cristatus* only in two different seasons, selected for a maximum contrast of climatic conditions. The *T*. *cristatus* were fed a mixed diet of fresh leaves, vegetables and commercial pellets (S2 Table) three times daily. The amounts of each feed were the same between trials across the seasons. Tree leaves harvested by the zoo staffs inside the zoological garden, were fed with branch and twigs once a day at noon, and the other feeds provided by commercial suppliers, were fed in the morning and evening.

We measured the body mass of all animals before and after the sampling periods in each experiment (*N*. *larvatus*: Digital Platform Scale, DP-8100, Yamato, Japan; *T*. *cristatus*: SD75LJP, OHAUS Corporation, USA), by offering weighing platforms on which the animals stepped voluntarily.

### Sampling procedures

Feed intake was quantitatively recorded over seven sampling days. Each food item was weighed before it was offered to the animals and left in their enclosures until the next feeding session. S1 and S2 Tables shows the mean (±standard error) daily amount of offered food per animal. All leftover food was removed, and the enclosure was cleaned before fresh food items were offered. All food items and leftovers were weighed with accuracy of 2 g (browse, UDS-500N, Yamato, Japan) or 1 g (others, UH-3201, A&D Company, Japan). Leftover weights were adjusted by deriving a desiccation factor from the measured moisture lost from similar sets of food placed in a desiccation pan in an area adjacent to the primate enclosures. For the two *T*. *cristatus* at Japan Monkey Centre housed together, individual feed intake and faeces output were calculated as the average of total measures divided by two.

We collected equal amounts of each feedstuff at every feeding time during the sampling period and stored them in a refrigerator (10°C: trials for *N*. *larvatus*; 4°C: trials for *T*. *cristatus*) and a freezer (-18 to 20°C: only leaves and banana peel for *T*. *cristatus*) for nutritional analysis. We also collected all faeces shortly before every feeding time and immediately preserved them in the freezer. We mixed each feed sample and then collected 100 g of leaves of each browse species and all amounts of other foods as representative samples. We mixed all faeces with 1 ml/100 g of 10% formalin solution and preserved 500 g faeces as a representative sample [20].

After measuring the fresh weight of representative samples, in the case of *N*. *larvatus*, we lyophilised leaf and faeces samples using a freeze-dryer (DC400/800, Yamato, Japan). For other feedstuff samples (vegetables and commercial pellets), we used an air-forced dry oven (DKM812, Yamato, Japan) for 48 h at 60°C. Note that we mashed soybean and peanuts in a mortar due to their high fat content and then soaked and washed the mixture with ether to extract fat. After fat extraction, the soybean and peanuts were dried using an air-forced dry oven (DKM600, Yamato, Japan) for 3 h at 60°C. In the case of *T*. *cristatus*, we dried all feedstuff and faecal samples using an air-forced dry oven (DKM812, Yamato, Japan) for 48 h at 60°C.

We ground leaf and faecal samples using a Wiley mill through a 2-mm screen and ground other feed samples using a coffee mill to avoid heat denaturation of sugars in the feeds.

### Estimation of apparent digestibility (aD)

We used the average daily feed intake and faecal output during the sampling term to estimate the aD in each trial. We analysed the dry matter (DM), crude ash (CA), crude protein (CP) and acid detergent fibre (ADFom) in accordance with AOAC 930.15, 942.05, 990.03 and 973.18, respectively [21]. We then analysed neutral detergent fibre (aNDFom) according to the method of van Soest, Robertson and Lewis [22]. Detergent fibre data are presented without residual ash. We calculated aD of each nutrient (N) according to the following equation: aD_N_ (%) = (N_feed intake_–N_feces_)/N_food intake_ × 100.

### Literature data and analysis

Together with our new experimental data, as shown in Table 1, we used published aD data of seven colobine species, including four tripartite species (18 datasets) [16,17,23–27] and two quadripartite species (five datasets) [17,18,28] to compare digestive capacity between the stomach groups. These published data included total feed and fibre intake and aD of DM, NDF and ADF, and for some but not all studies the body mass of the animals used.

**Table 1:** Summary of colobine apparent digestibility and body mass in the previous studies.

Colobus monkey		Body mass (kg)	Feed stuff				Apparent digestibility			Reference
Stomach compartments	species		Browse	Fruits Vegetable	Artificial Food	Others*^1^	DM	NDF	ADF	
3	*Colobus guereza*	10.9			✓ (15ADF)		80.3	77.0	80.1	Edwards and Ullrey [17]
		10.7			✓ (30ADF)		78.7	74.3	56.2	
		7.0 *^2^	✓	✓	✓		65	56	56	Nijboer [26]
			✓	✓	✓		68	63	56	
		8.9		✓	✓		83 (82–84)	68	68	Oftedal, Jakubasz and Whetter [24]
		8.0		✓	✓	✓	87.1 ± 0.1	81.3 ± 1.0	69.3 ± 0.5	Watkins, Ullrey and Whetter [25]
	*Trachypithecus francoisi*	6.0			✓ (15ADF)		82.4±3.0	79.3 ± 5.3	82.3 ± 4.8	Edwards and Ullrey [17]
		6.0			✓ (30ADF)		76.7 ± 3.5	75.7 ± 7.7	76.9 ± 12.8	
		-	✓	✓	✓		58.9	46.6	38.5	Nijboer et al. [23]
			✓	✓	✓		64.7	62.2	56.1	
			✓	✓	✓		74.0	68.0	65.3	
	*Trachypithecus auratus*	6.0	✓	✓	✓	✓	68	-	-	Nijboer et al. [27]; Nijboer [26]
			✓		✓	✓	59	-	-	
	*Trachypithecus cristatus*	-			✓		75.7 ± 5.5	68.9 ± 5.8	61.6 ± 7.5	Sakaguchi et al. [16]
	*Trachypithecus obscurus*	6.0 *^2^	✓	✓	✓	✓	91	70	53	Nijboer [26]
			✓	✓	✓	✓	84	77	54	
			✓	✓	✓	✓	78	74	65	
			✓	✓	✓	✓	78	64	60	
4	*Nasalis larvatus*	9.0 *^2^	✓	✓	✓	✓	88.3	86.4	85.0	Dierenfeld, Koontz and Goldstein [28]
			✓	✓	✓	✓	88.8	86.2	86.0	
	*Pygathrix nemaeus*	12.1			✓ (15ADF)		76.2	66.5	66.6	Edwards and Ullrey [17]
		11.75			✓ (30ADF)		73.9	69.8	67.6	
		-	✓	✓			-	89.2	83.9	Coudrat and Cabana [18]

*1 Others containing non-fibrous carbohydrate (rice, bread, sweet potato, cereal and Saint John’s bread), animal protein (boiled egg, meat and mealworm) and vegetable protein (tofu).

*2 Median of specimens used for digestive trials.

Due to the limited data, we only focussed on two-factorial models and did not perform models with more variables/factors. First, we tested two-factorial linear models that linked the aD to either combination of body mass, stomach type and NDF content of the ingested diet. Second, in the larger dataset (including the studies did not provide body mass information), we examined the relationship between aD in DM, NDF and ADF and the dietary NDF content in each colobine group with different stomach type to estimate the slope and intercept using linear regression analysis. Analysis of covariance served to compare the slopes and intercepts of regression lines between the stomach types. Note that we could not conduct the same statistical analysis for the aD of CP because of the lack of published data. Additionally, because Nijboer [26] and Nijboer et al. [27] in *Trachypithecus auratus* analysed only crude fibre as fibre contents and Coudrat and Cabana [18] did not specify DM contents in diets and faeces with the total intake (see Table 1), we could not include these data in our analysis. All statistical analyses were performed in Spyder (Python 3.7).

## Results

### Digestion trials

The body mass of *Nasalis larvatus* in autumn, winter and summer was 17.3, 18.9 and 17.8 kg, respectively, shortly before beginning of the sampling periods. The composition and amount of the diet were different amongst trials (S1 Table). The ratio of leaves/other feed intake were 7.4:1, 3.3:1 and 3.5:1, in autumn, winter and summer. Browse species generally contained more fibre than other feeds (S3–S5 Tables), possibly leading to different proportion of ADFom in the diet in autumn, i.e. 33.3%, 29.4% and 30.1% of DM in autumn, winter and summer, respectively. There was notable seasonal variation in the nutritional composition of the browse species (S3–S5 Tables). The DM and ash contents of laurel (*Machilus thunbergii*) in winter were 5%–10% higher than those in summer and winter. The aNDFom and ADFom of each browse species in winter were 5%–10% lower than those in summer and autumn. On the other hand, nutrient contents of fruits, vegetables, beans, starchy foods and commercial products in *N*. *larvatus* did not differ between the seasons (S3–S5 Tables). The aD of DM was 69.9%, 79.6% and 73.7% in autumn, winter and summer, respectively (Table 2). Likewise, the aD of CP, aNDFom and ADFom varied amongst the three seasons (Table 2).

**Table 2:** DM intake, faecal output and apparent digestibility (aD) in *Nasalis larvatus* and *Trachypithecus cristatus*.

	*Nasalis larvatus*			*Trachypithecus cristatus*	
	2017 Sep (Autumn)	2018 Jan (Winter)	2018 Jun (Summer)	2018 Aug (Summer)	2019 Feb (Winter)
Body mass (kg)	17.3	18.9	17.8	7.6/7.6	6.8/7.7
DM intake (g DM/day/animal)	702.0	654.9	664.3	146.0	133.3
Fecal output (g DM/day/animal)	172.3	109.1	102.8	17.6	16.4
Ingested diet					
OM (%DM)	91.3	90.6	92.6	93.8	93.4
CP (%DM)	11.7	11.7	11.9	7.3	7.9
αNDFom %DM)	37.9	35.3	37.4	12.9	15.0
ADFom (%DM)	33.3	29.1	30.1	10.3	12.7
Feces					
OM (%DM)	89.3	88.5	87.4	88.4	88.3
CP (%DM)	17.3	14.9	19.0	26.3	25.0
αNDFom %DM)	39.4	41.4	34.5	31.1	30.0
ADFom (%DM)	34.7	40.2	39.8	23.3	28.2
aD DM (%)	75.5	83.0	84.5	87.9	87.7
aD OM (%)	76.0	83.7	85.4	88.6	88.3
aD CP (%)	63.8	78.8	75.3	56.4	61.1
aD αNDFom (%)	74.5	80.4	85.7	70.8	75.4
aD ADFom (%)	74.4	77.0	79.5	72.7	72.6

DM, dry matter; OM, organic matter; CP, crude protein; aNDFom, neutral detergent fibre; ADFom, acid detergent fibre.

The body mass of the two *T*. *cristatus* was 7.6 and 7.6 kg in summer and 6.8 and 7.7 kg in winter shortly before beginning of the sampling period. There was strong seasonality in the nutritional composition of their browse (S6 and S7 Tables). The DM and ash contents in the browse species in *T*. *cristatus* were higher, and CP, aNDFom and ADFom were lower in winter than in summer. The other diet items, i.e. fruits,vegetables, starchy foods and commercial product, did not differ between the two seasons (S6 and S7 Tables). The aD of DM, ash and ADFom did not differ between the two seasons; only the aD of CP and aNDFom changed between the two seasons (Table 2).

### Literature data

#### Apparent digestibility

Body mass differed significantly between species with a tripartite (mean = 7.39 kg; sd = 0.71) or a quadripartite (13.7 kg; sd = 4.23) stomach (Z = 3.40, p < 0.001).

In a model linking aD DM with body mass and stomach type (R^2^ = 0.016, p = 0.862), neither body mass (F = 0.015, p = 0.903) nor stomach type (F = 0.072, p = 0.792) were significant. However, for aD NDF and aD ADF, particularly stomach type approached significance in both models, and body mass in the model for aD ADF (whole model: R^2^ = 0.170, p = 0.187 and R^2^ = 0.252, p = 0.074; body mass F = 2.252, p = 0.150 and F = 3.025, p = 0.074; stomach type: F = 3.866, p = 0.064 and F = 3.025, p = 0.074, respectively).

On the other hand, in a model linking aD DM with body mass and NDF contents of the ingested diet (R^2^ = 0.571, p = 0.0005), only the NDF content (F = 22.04, p < 0.001) was significant but not body mass (F = 0.015, p = 0.903). For aD NDF and aD ADF, the neither body mass nor NDF content were significant (whole model: R^2^ = 0.287, p = 0.047 and R^2^ = 0.364, p = 0.017; body mass: F = 2.252, p = 0.150 and F = 2.998, p = 0.100; NDF contents: F = 2.138, p = 0.160 and F = 2.744, p = 0.114, respectively). Note that in comparison to the models presented further below, sample sizes were limited as not all literature provided data on body mass.

The final model for the limited dataset evaluated the effects of aD DM on the stomach type and NDF content (R^2^ = 0.550, p = 0.001), where only the NDF content (F = 22.04, p = 0.0002) was significant but not stomach type (F = 0.072, p = 0.792). For aD NDF and aD ADF, only stomach type was significant or approached significance (whole model: R^2^ = 0.291, p = 0.045 and R^2^ = 0.406, p = 0.009; stomach type: F = 3.866, p = 0.064 and F = 6.382, p = 0.021; NDF contents: F = 2.138, p = 0.160 and F = 2.744, p = 0.114, respectively).

**Fig 1:**
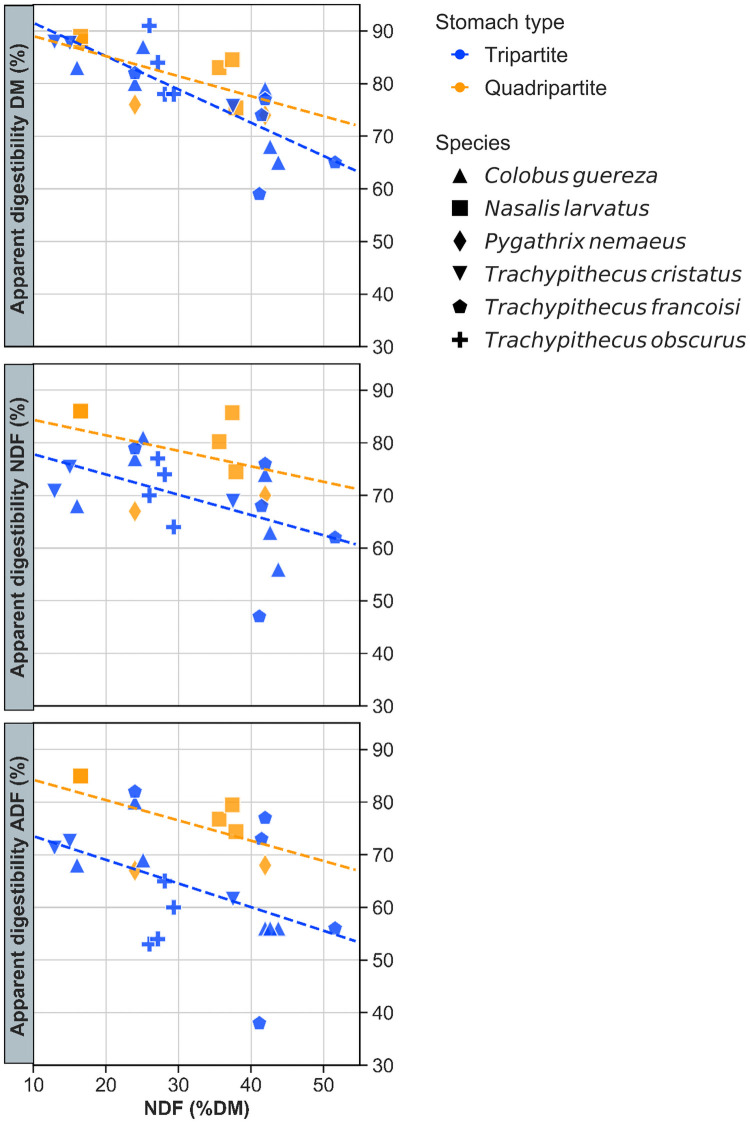
Relationship between apparent digestibility of dry matter (DM, %), neutral detergent fibre (NDF, %) or acid detergent fibre (ADF, %) and neutral detergent fibre intake (%) in six colobine species: *Colobus guereza* [17,24–26], *Nasalis larvatus* [28 and this study], *Pygathrix nemaeus* [17], *Trachypithecus cristatus* [16 and this study], *Trachypithecus francoisi* [17,23] and *Trachypithecus obscurus* [26]. Species and stomach type, i.e. tri- or quadripartite, are indicated in different shape and colour symbols, respectively.

In the larger dataset, there was a significant negative relationship between the aD of DM and the NDF content of the ingested diet of six colobine species (r = −0.770, p < 0.001). The difference in the regression slopes and intercepts for aD of DM vs. NDF content of the ingested diet between the stomach types was not significant (Fig 1; slope, t = 1.079; p = 0.293; intercept, t = 1.092, p = 0.287). There was also a significant negative relationship between the aD of NDF or the aD of ADF and the NDF content of the ingested diet of six colobine species (NDF, r = −0.459, p = 0.021; ADF, r = −0.439, p = 0.028). The differences in the regression intercepts for both aD of NDF and aD of ADF vs. NDF content were significant (NDF, t = −2.559, p = 0.018; ADF, t = −2.487, p = 0.021) between the stomach types (Fig 1), while the slopes were not different (NDF, t = 0.267, p = 0.792; ADF, t = 0.152, p = 0.881).

## Discussion

As expected, we confirmed the variation in the aD because of the nutrient differences in the diets across the seasons. Each browse species’ nutrient composition changed seasonally, as reported for deciduous tree species in North American zoos [14], whereas those of commercial food items were stable (S3–S7 Tables). The aD of DM, aNDFom and ADFom in *N*. *larvatus* clearly changed across seasons: aD DM by 9.1%, aD aNDFom by 11.2% and aD ADFom by 5.1%. On the other hand, aD was relatively stable between the two different seasons in *T*. *cristatus*, although the total DM intake in winter was slightly higher than that in summer. As in the present study, Edwards and Ullrey [17] noted that the aD of DM in three colobine species (*Colobus guereza*, *Pygathrix nemaeus* and *Trachypithecus francoisi*) decreased with an increased fibre level in the diet, supporting that aD is affected by a variation in food composition and seasonally varying nutrient contents in feeds. Consequently, the aD of *N*. *larvatus* had a larger seasonal variation, with more distinctively varying nutrient intake across seasons, than that of *T*. *cristatus*, which mostly consumed commercial foods with a lower proportion of browse. Evaluating the nutrient digestibility of captive colobines on different diets throughout the year may contribute to their health management and predict intake requirements across different diets and seasons.

Comparison of the nutrient composition (especially fibre) of faeces in free-ranging and captive individuals has been proposed to obtain information relevant for the improvement of diets of colobines [10]. *Nasalis larvatus* in our study had faecal NDF contents (35%–41% in DM) that were higher than those reported in other captive conspecifics, i.e. 17% [28] (mean of two different values), but lower than those of free-ranging ones, i.e. 53%–70% [10]. Although faecal NDF contents of free-ranging *T*. *cristatus* are not available, those in our study (30%–31%) were comparable to other closely related species in captivity, i.e. 31% (mean of three different values in *T*. *francoisi*) [23,26], although still far lower than those reported for free-ranging *N*. *larvatus*. Altering the diets of captive colobines to include more fibre, comparable to those of free-ranging ones, may be recommendable.

**Fig 2:**
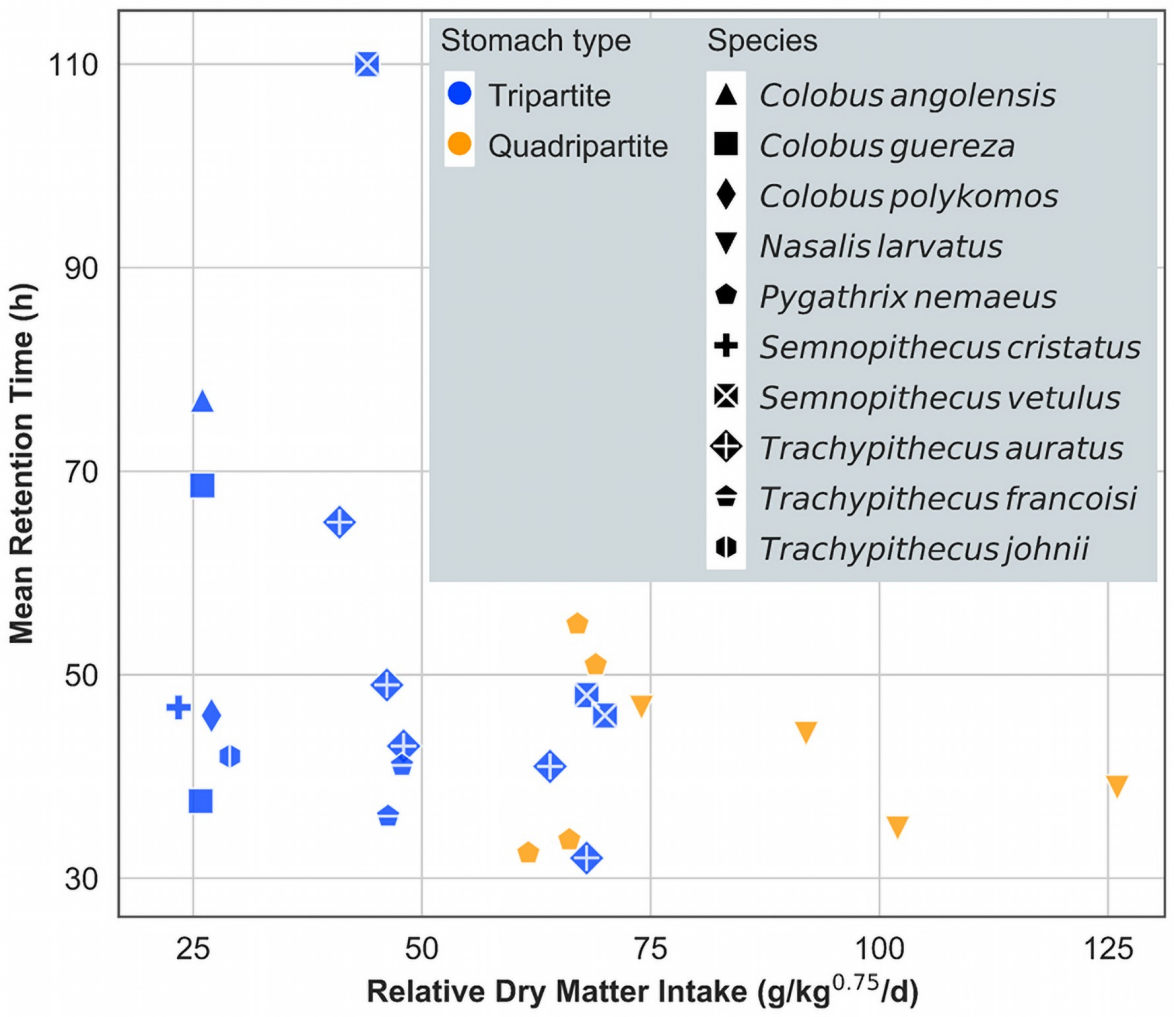
Relationship between mean retention time (h) and relative dry matter intake (g/kg^0.75^/d) in 10 colobine species: *Colobus angolensis* [31], *Colobus guereza* [17], *Nasalis larvatus* [19], *Pygathrix nemaeus* [17], *Semnopithecus cristatus* [16], *Semnopithecus vetulus* [32], *Trachypithecus auratus* [27], *Trachypithecus francoisi* [17] and *Trachypithecus johnii* [31]. Species and stomach type, i.e. tri- or quadripartite, are indicated in different shape and colour symbols, respectively.

In the present study, the NDF level of the total DM intake was much higher in the quadripartite species *N*. *larvatus* (35.6%–37.9%) than in the tripartite species *T*. *cristatus* (12.9%–15.0%), because of a much higher proportion of browse fed to the former. This zoo practice may stem from the impression that quadripartite species are generally more difficult to maintain in captivity; therefore, more effort is undertaken to provide them with feed items considered natural for them, mainly browse. Correspondingly, we observed a higher DM intake (% BW, shown in S3–S5 Tables) in *N*. *larvatus* compared with those in *T*. *cristatus* that could be interpreted as compensation for the higher fibre levels. However, regardless of the higher fibre and intake levels, we observed higher aD in DM, aNDFom and ADFom (Table 2) in the *N*. *larvatus* specimen. This observation is most parsimoniously explained by a higher gut capacity in the proboscis monkey, provided by the additional praesaccus forestomach chamber. Typically, a higher relative food intake leads to shorter digesta retention times and can also compromise digestibility [29,30, cf. also Fig 2]. However, a higher gut capacity can mitigate this effect, and this may be the main adaptive value of the praesaccus in quadripartite species [15].

Unfortunately, the currently available information for tri- and quadripartite species is biased in terms of the body mass of the investigated specimens. Although the overall distribution of tri- and quadripartite stomach types across colobine genera [15] does not suggest a body size effect on stomach anatomy, quadripartite specimens of the literature data collection were significantly heavier than tripartite specimens. This is due to the inclusion of *N*. *larvatus*–the largest colobine, with a quadripartite stomach. Given an older but prevailing assumption in the ecophysiological literature that body mass is positively related to digesta retention time and digestibility [33], this could lead to the suspicion that differences between the stomach types are body size effects. However, this is refuted on the one hand by our own analyses that showed no effect of body mass or a trend subordinate to the trend of stomach type, and on the other hand by more recent literature. An effect of body size on digesta retention and digestion has been contested by various large empirical data collections as well as by theoretical considerations [30,34,35], including exclusive analyses of primate data.

The results of our analysis for aD using the larger literature data set further suggested functional differences between the stomach types. There was no difference in how dietary fibre content influenced the overall DM digestibility, which is, in most of the diets used in these studies, most likely dominated by the digestion of non-fibrous carbohydrates and protein. However, stomach type had a significant effect on how dietary fibre levels affected the digestibility of fibre itself. Here, quadripartite species achieved higher fibre digestibility at similar dietary fibre levels than tripartite species, suggesting that the additional gut capacity offered by the praesaccus facilitates longer retention and hence more thorough microbial fermentation of plant fibre. One notable tendency was that *N*. *larvatus* achieved a particularly high digestibility, driving the difference between the two stomach types. In this species, regurgitation and remastication have been observed in the wild [36], and a smaller faecal particle size of *N*. *larvatus* compared with other colobines has been reported [37]. Because particle size reduction via chewing is one of several key factors affecting digestibility [38], we cannot determine whether the difference in digestibility observed here is related to stomach type or chewing efficiency. Ideally, in future studies, similar diets should be employed as this will allow meaningful comparison of faecal particle size; chewing behaviour should also be observed.

Unfortunately, the current available data on digesta retention times in colobine species does not allow testing for a general difference between tripartite and quadripartite species (Fig 2). Between species, retention times need to be compared in relation to the food intake level [29,39]. However, the ranges of intake level in published studies hardly overlap between the stomach groups, making a reasonable comparison impossible. To test whether quadripartite species achieve longer digesta retention because of a higher gut fill, comparative studies with different species on a similar (possibly browse-dominated) diet would be required where intake, digestibility and digesta retention are assessed in the same experiment, additionally facilitating the calculation of gut fill [40,41]. Ideally, such a study would also address the problem of the very limited sample size of the present experiments. Until such a study is performed, our results must be considered preliminary, delivering plausible hypotheses.

It should be noted that we cannot exclude the possibility of the effects of specific fibre-digesting bacteria in the presaccus in quadripartite species. So, far, only a few analyses of the forestomach microbiome are available for colobines. Although the recent developments in sequencing technology describe the foregut microbiome in some colobines, e.g. *N*. *larvatus* [42,43] and *Rhinopithecus roxellana* [44], the function of these microbe species has not been evaluated. However, there is currently also no reason to assume that the praesaccus should harbour a fundamentally different microbiome from the saccus. Detailed studies about differences in the microbiome at different forestomach locations, as available in ruminants [e.g. 45], do not exist for colobines so far. In contrast to the detailed knowledge about the differential function of individual forestomach sections in ruminants and camelids [e.g. 46], there is, to date, no indication of the differential function of the forestomach compartments of non-ruminant foregut fermenters [47] beyond the provision of sheer fermentation chamber capacity, and this may well also apply to colobines.

## Supporting information

S1 Table Feed composition, and offered and leftover amounts (g as fed/day) in each digestion trial of an adult male of *Nasalis larvatus*. *^1^ Weight unit is X ± SE g, as fed/day/animal. *^2^ We measured the weight of the whole branch, including leaves and twigs.
(TIF)

S2 Table Feed composition, and offered and leftover amounts (g as fed/day/two animals) in each digestion trial of two adults of *Trachypithecus cristatus*. *1 Weight unit is X ± SE g FM/day. *2 We measured the weight of the whole branch, including leaves and twigs.
(TIF)

S3 Table Nutrient contents in the feeds and DM intake of an adult male of *Nasalis larvatus* in September 2017 (autumn). DM, dry matter; ash, crude ash; CP, crude protein; aNDFom, neutral detergent fibre assayed with a heat stable amylase exclude residual ash; ADFom, acid detergent fibre excluded residual ash; DMI, dry matter intake; BW, body mass (kg). *1 Intake/offered of each feed (DM-basis). Browses were weighed as the whole branch, including leaves and twigs, but nutrient contents were analysed only for leaves. Thus, we could not calculate offered DM amounts of browse. *2 DM intake of each feed/total DM intake. *3 BW of the proboscis monkey was 17.3 kg at the beginning of the second week of the experiment.
(TIF)

S4 Table Nutrient contents in the diet and DM intake of an adult male of *Nasalis larvatus* in January 2018 (winter). DM, dry matter; ash, crude ash; CP, crude protein; aNDFom, neutral detergent fibre assayed with a heat stable amylase exclude residual ash; ADFom, acid detergent fibre excluded residual ash; DMI, dry matter intake; BW, body mass (kg). *1 Intake/offered of each feed (DM-basis). Browses were weighed as the whole branch, including leaves and twigs, but nutrient contents were analysed only for leaves. Thus, we could not calculate offered DM amounts of browse. *2 DM intake of each feed/total DM intake. *3 BW of the proboscis monkey was 18.9 kg.at the beginning of the second week of the experiment. *4 The pelleted feed was soaked in water before feeding.
(TIF)

S5 Table Nutrient contents in the diet and DM intake of an adult male of *Nasalis larvatus* in June 2018 (summer). DM, dry matter; ash, crude ash; CP, crude protein; aNDFom, neutral detergent fibre assayed with a heat stable amylase exclude residual ash; ADFom, acid detergent fibre excluded residual ash; DMI, dry matter intake; BW, body mass (kg). *1 Intake/offered of each feed (DM-basis). Browses were weighed as the whole branch, including leaves and twigs, but nutrient contents were analysed only for leaves. Thus, we could not calculate offered DM amounts of browse. *2 DM intake of each feed/total DM intake. *3 BW of the proboscis monkey was 17.8 kg at the beginning of the second week of the experiment.
(TIF)

S6 Table Nutrient contents in the diet and DM intake of two adults *Trachypithecus cristatus* in August 2018 (summer). DM, dry matter; ash, crude ash; CP, crude protein; aNDFom, neutral detergent fibre assayed with a heat stable amylase exclude residual ash; ADFom, acid detergent fibre excluded residual ash; DMI, dry matter intake; BW, body mass (kg). *1 Intake/offered of each feed (DM-basis). Browses were weighed as the whole branch, including leaves and twigs, but nutrient contents were analysed only for leaves. Thus, we could not calculate offered DM amounts of browse. *2 DM intake of each feed/total DM intake. *3 BW of two silver lutungs were 7.6 and 7.6 kg at the beginning of the second week of the experiment.
(TIF)

S7 Table Nutrient contents in the diet and DM intake of two adults of *Trachypithecus cristatus* in February 2019 (winter). DM, dry matter; ash, crude ash; CP, crude protein; aNDFom, neutral detergent fibre assayed with a heat stable amylase exclude residual ash; ADFom, acid detergent fibre excluded residual ash; DMI, dry matter intake; BW, body mass (kg). *1 Intake/offered of each feed (DM-basis). Browses were weighed as the whole branch, including leaves and twigs, but nutrient contents were analysed only for leaves. Thus, we could not calculate offered DM amounts of browse. *2 DM intake of each feed/total DM intake. *3 BW of two silver lutungs were 6.8 and 7.7 kg at the beginning of the second week of the experiment.
(TIF)

## References

[1] Campbell CJ, Fuentes A, MacKinnon KC, Bearder SK, Stumpf RM. Primates in perspective. edition S, editor. New York: Oxford University Press; 2011.

[2] Lambert JE. Primate digestion: Interactions among anatomy, physiology, and feeding ecology. Evolutionary Anthropology: Issues, News, and Reviews. 1998;7(1):8–20.

[3] Chivers D. Functional anatomy of the gastrointestinal tract. In: Davies A, Oates J, editors. Colobine monkeys: their ecology, behaviour and evolution. Cambridge: Cambridge University Press; 1994. p. 205–27.

[4] Bauchop T, Martucci RW. Ruminant-like digestion of the langur monkey. Science. 1968;161(3842):698–700. doi: 10.1126/science.161.3842.698 4969750

[5] Clauss M, Dierenfeld ES. The nutrition of browsers. In: Fowler ME, Miller RE, editors. Zoo and Wild Animal Medicine: Current Therapy. 6 ed. St. Louis: Saunders Elsevier; 2008. p. 444–54.

[6] Matsuda I, Clauss M. Morphology and physiology of colobine digestive tracts. In: Matsuda I, Grueter CC, Teichroeb JA, editors. The Colobines: Natural History, Behaviour and Ecological Diversity. Cambridge: Cambridge University Press; in press.

[7] Langer P. The mammalian herbivore stomach. Stuttgart/New York: Gustav Fischer Verlag; 1988.

[8] Langer P. Comparative anatomy of the gastrointestinal tract in Eutheria: Taxonomy, biogeography and food. Vol I: Afrotheria, Xenarthra and Euarchontoglires. Vol II: Laurasiatheria, general discussion. Zachos FE, editor. Berlin: De Gruyter; 2017.

[9] Caton JM. The morphology of the gastrointestinal tract of *Pygathrix nemaeus*. In: Jablonski NG, editor. Natural history of the doucs and snub-nosed monkeys. Singapore: World Scientific; 1998. p. 129–49.

[10] Matsuda I, Bernard H, Tuuga A, Nathan S, Sha JCM, Osman I, et al. Fecal nutrients suggest diets of higher fiber levels in free-ranging than in captive proboscis monkeys (*Nasalis larvatus*). Front Vet Sci. 2017;4:246. doi: 10.3389/fvets.2017.00246. 29404345 PMC5780573

[11] Struhsaker TT. The Red Colobus Monkeys: Variation in Demography, Behavior, and Ecology of Endangered Species. New York: Oxford University Press; 2010.

[12] Hollihn UWE. Remarks on the breeding and maintenance of Colobus monkeys *Colobus guereza*, Proboscis monkeys *Nasalis larvatus* and Douc langurs *Pygathrix nemaeus* in zoos. International Zoo Yearbook. 1973;13(1):185–8. doi: 10.1111/j.1748-1090.1973.tb02146.x

[13] Lhota S, Sha JCM, Bernard H, Matsuda I. Proboscis monkey conservation: beyond the science. In: Behie AM, Teichroeb JA, Malone NM, editors. Primate Research and Conservation in the Anthropocene. Cambridge Cambridge University Press; 2019. p. 182–96.

[14] Nijboer J, Dierenfeld ES. Comparison of diets fed to southeast Asian colobines in North American and European zoos, with emphasis on temperate browse composition. Zoo Biology. 1996;15(5):499–507.

[15] Matsuda I, Chapman CA, Clauss M. Colobine forestomach anatomy and diet. J Morphol. 2019;280(11):1608–16. doi: 10.1002/jmor.21052 . 31424606

[16] Sakaguchi E, Suzuki K, Kotera S, Ehara A, editors. Fibre digestion and digesta retention time in Macaque and Colobus monkeys. XIIIth congress of the Int Primatolgy Society; 1991: Elsevier.

[17] Edwards MS, Ullrey DE. Effect of dietary fiber concentration on apparent digestibility and digesta passage in non-human primates. II. Hindgut- and foregut-fermenting folivores. Zoo Biology. 1999;18(6):537–49.

[18] Coudrat C, Cabana F. Preliminary results on the food intake and nutrient digestibility of southern white-cheeked gibbons (*Nomascus siki*) and red-shanked douc langurs (*Pygathrix nemaeus*) at the Endangered Primate Rescue Center, Vietnam. Vietnamese Journal of Primatology. 2019;3(1):71–6.

[19] Matsuda I, Sha JC, Ortmann S, Schwarm A, Grandl F, Caton J, et al. Excretion patterns of solute and different-sized particle passage markers in foregut-fermenting proboscis monkey (*Nasalis larvatus*) do not indicate an adaptation for rumination. Physiol Behav. 2015;149:45–52. doi: 10.1016/j.physbeh.2015.05.020 . 26004169

[20] Ishida M. Digestive trial for ruminants. In: Ishibashi T, editor. Experimental Methods for Animal Nutrition (in Japanese). Tokyo: Yokendo; 2001. p. 190–7.

[21] AOAC. Official Methods of Analysis of AOAC International. Gaithersburg, MD: Association of Official Analytical Chemists International; 2012.

[22] van Soest PJ, Robertson JB, Lewis BA. Methods for Dietary Fiber, Neutral Detergent Fiber, and Nonstarch Polysaccharides in Relation to Animal Nutrition. Journal of Dairy Science. 1991;74(10):3583–97. doi: 10.3168/jds.S0022-0302(91)78551-2 1660498

[23] Nijboer J, Becher F, van der Kuilen J, Beynen AC. Chemical analysis and consistency of faeces produced by captive monkeys (*Francois langurs*, *Trachypithecus francoisi*) fed supplemental fibre. Veterinary Quarterly. 2001;23(2):76–80. doi: 10.1080/01652176.2001.9695086 . 11361103

[24] Oftedal OT, Jakubasz M, Whetter P. Food intake and diet digestibility by captive black and white colobus (*Colobus guereza*) at the National Zoological Park. Ann Proc Amer Assoc Zoo Vet. 1982;33:(Abstract).

[25] Watkins BE, Ullrey DE, Whetter PA. Digestibility of a high-fiber biscuit-based diet by black and white colobus (*Colobus guereza*). American Journal of Primatology. 1985;9(2):137–44. doi: 10.1002/ajp.1350090207 32102487

[26] Nijboer J. Fibre intake and faeces quality in leaf-eating primates: Utrecht University; 2006.

[27] Nijboer J, Clauss M, van de Put K, van der Kuilen J, Woutersee H, Beynen AC. Influence of two different diets on fluid and particle retention time Javan langur (*Trachypithecus auratus auratus*). Der Zoologische Garten. 2007;77(1):36–46. doi: 10.1016/j.zoolgart.2007.06.004

[28] Dierenfeld ES, Koontz FW, Goldstein RS. Feed intake, digestion and passage of the proboscis monkey (*Nasalis larvatus*) in captivity. Primates. 1992;33(3):399–405. doi: 10.1007/bf02381201

[29] Clauss M, Streich WJ, Nunn CL, Ortmann S, Hohmann G, Schwarm A, et al. The influence of natural diet composition, food intake level, and body size on ingesta passage in primates. Comp Biochem Physiol A Mol Integr Physiol. 2008;150(3):274–81. doi: 10.1016/j.cbpa.2008.03.012 . 18450489

[30] Müller DW, Codron D, Meloro C, Munn A, Schwarm A, Hummel J, et al. Assessing the Jarman-Bell Principle: Scaling of intake, digestibility, retention time and gut fill with body mass in mammalian herbivores. Comp Biochem Physiol A Mol Integr Physiol. 2013;164(1):129–40. doi: 10.1016/j.cbpa.2012.09.018 . 23047052

[31] Schwarm A, Ortmann S, Wolf C, Streich WJ, Clauss M. Passage marker excretion in red kangaroo (*Macropus rufus*), collared peccary (*Pecari tajacu*) and colobine monkeys (*Colobus angolensis*, *C*. *polykomos*, *Trachypithecus johnii*). Journal of experimental zoology Part A, Ecological genetics and physiology. 2009;311(9):647–61. doi: 10.1002/jez.552 . 19551808

[32] Matsuda I, Espinosa-Gomez FC, Ortmann S, Sha JCM, Osman I, Nijboer J, et al. Retention marker excretion suggests incomplete digesta mixing across the order primates. Physiol Behav. 2019;208:112558. doi: 10.1016/j.physbeh.2019.112558. 31125579

[33] Demment MW, Van Soest PJ. A nutritional explanation for body-size patterns of ruminant and nonruminant herbivores. The American Naturalist. 1985;125(5):641–72. doi: 10.1086/284369

[34] Clauss M, Steuer P, Muller DW, Codron D, Hummel J. Herbivory and body size: allometries of diet quality and gastrointestinal physiology, and implications for herbivore ecology and dinosaur gigantism. PLoS One. 2013;8(10):e68714. doi: 10.1371/journal.pone.0068714. 24204552 PMC3812987

[35] Steuer P, Südekum K-H, Tütken T, Müller DWH, Kaandorp J, Bucher M, et al. Does body mass convey a digestive advantage for large herbivores? Functional Ecology. 2014;28(5):1127–34. doi: 10.1111/1365-2435.12275

[36] Matsuda I, Murai T, Clauss M, Yamada T, Tuuga A, Bernard H, et al. Regurgitation and remastication in the foregut-fermenting proboscis monkey (*Nasalis larvatus*). Biology letters. 2011;7(5):786–9. doi: 10.1098/rsbl.2011.0197 . 21450728 PMC3169055

[37] Matsuda I, Tuuga A, Hashimoto C, Bernard H, Yamagiwa J, Fritz J, et al. Faecal particle size in free-ranging primates supports a ’rumination’ strategy in the proboscis monkey (*Nasalis larvatus*). Oecologia. 2014;174:1127–37. doi: 10.1007/s00442-013-2863-9 . 24380969

[38] Hummel J, Clauss M, Südekum K-H. Aspects of food comminution in ungulates and their consequences for energy budget. In: Martin T, von Koenigswald W, editors. Mammalian teeth—form and function. Munich: Dr. Friedrich Pfeil; 2020.

[39] Levey DJ, Martinez del Rio C. Test, rejection, and reformulation of a chemical reactor-based model of gut function in a fruit-eating bird. Physiol Biochem Zool. 1999;72(3):369–83. doi: 10.1086/316663 . 10222331

[40] Munn AJ, Tomlinson S, Savage T, Clauss M. Retention of different-sized particles and derived gut fill estimate in tammar wallabies (*Macropus eugenii*): physiological and methodological considerations. Comp Biochem Physiol A Mol Integr Physiol. 2012;161(2):243–9. doi: 10.1016/j.cbpa.2011.11.003 . 22094100

[41] Munn A, Stewart M, Price E, Peilon A, Savage T, Van Ekris I, et al. Comparison of gut fill in sheep (*Ovis aries*) measured by intake, digestibility, and digesta retention compared with measurements at harvest. Canadian Journal of Zoology. 2015;93(10):747–53. doi: 10.1139/cjz-2014-0314

[42] Hayakawa T, Nathan S, Stark DJ, Saldivar DAR, Sipangkui R, Goossens B, et al. First report of foregut microbial community in proboscis monkeys: are diverse forests a reservoir for diverse microbiomes? Environ Microbiol Rep. 2018;10(6):655–62. doi: 10.1111/1758-2229.12677 . 29992728

[43] Suzuki-Hashido N, Tsuchida S, Hayakawa T, Sakamoto M, Azumano A, Seino S, et al. *Lactobacillus nasalidis* sp. nov., isolated from the forestomach of a captive proboscis monkey (*Nasalis larvatus*). Int J Syst Evol Microbiol. 2021;71(4). doi: 10.1099/ijsem.0.004787. 33906706

[44] Zhou X, Wang B, Pan Q, Zhang J, Kumar S, Sun X, et al. Whole-genome sequencing of the snub-nosed monkey provides insights into folivory and evolutionary history. Nat Genet. 2014;46(12):1303–10. doi: 10.1038/ng.3137 . 25362486

[45] Zeitz JO, Ineichen S, Soliva CR, Leiber F, Tschuor A, Braun U, et al. Variability in microbial population and fermentation traits at various sites within the forestomach and along the digestive tract as assessed in goats fed either grass or browse. Small Ruminant Research. 2016;136:7–17. doi: 10.1016/j.smallrumres.2015.12.029

[46] Idalan N, Martin LF, Clauss M. Physical characteristics of gastrointestinal content of llama (*Lama glama*). J Anim Physiol Anim Nutr (Berl). 2019;103(4):1015–22. doi: 10.1111/jpn.13116 . 31050031

[47] Schwarm A, Ortmann S, Fritz J, Flach E, Rietschel W, Clauss M. No distinct stratification of ingesta particles and no distinct moisture gradient in the fore-stomach of non-ruminants: The wallaby, peccary, hippopotamus, and sloth. Mammalian Biology—Zeitschrift für Säugetierkunde. 2013;78(6):412–21. doi: 10.1016/j.mambio.2013.04.001

