# Supplementary figures and images for: Apparent diet digestibility of captive colobines in relation to stomach types with special reference to fibre digestion
Source: PLoS One. 2021 Sep 20;16(9):e0256548. doi: 10.1371/journal.pone.0256548 (PMC8452005; doi:10.1371/journal.pone.0256548)

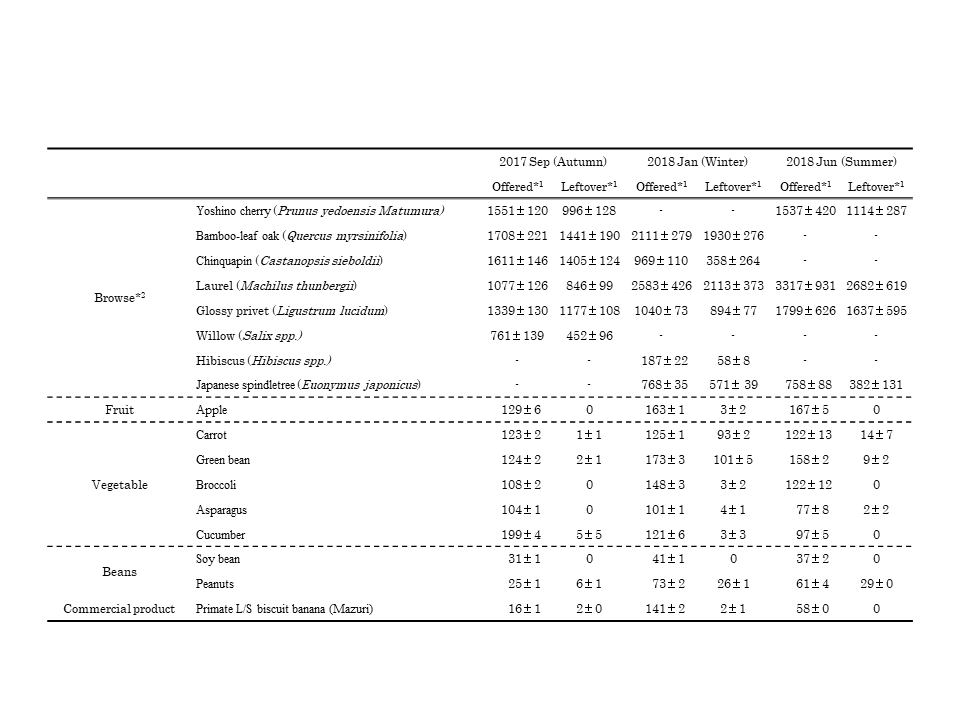

Supplement: S1 Table — *1 Weight unit is X ± SE g, as fed/day/animal. *2 We measured the weight of the whole branch, including leaves and twigs. (TIF) [file pone.0256548.s001.tif]

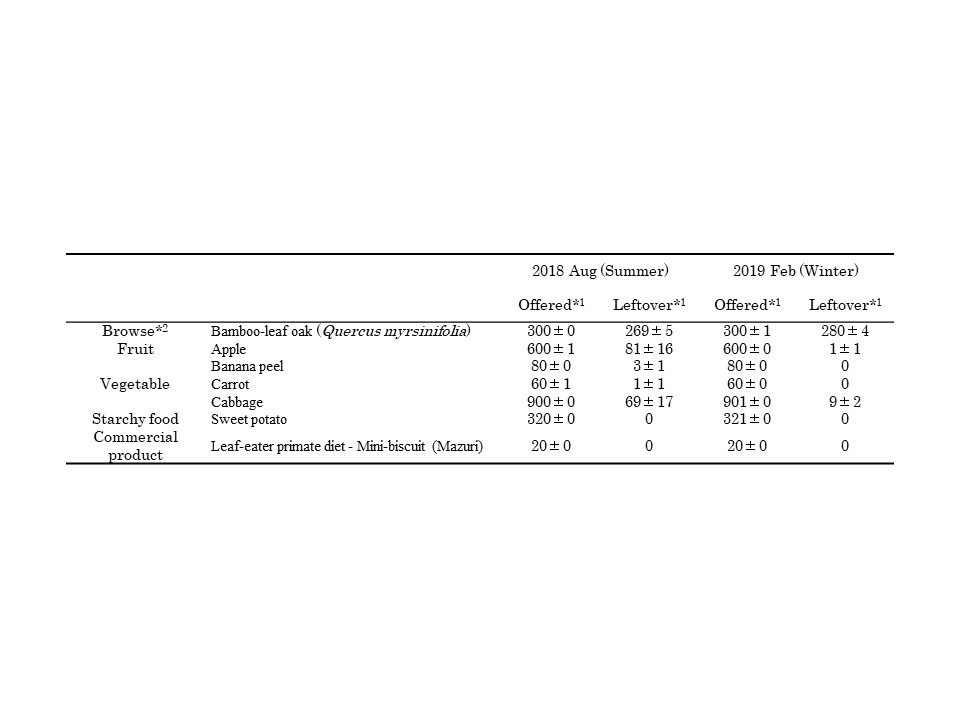

Supplement: S2 Table — *1 Weight unit is X ± SE g FM/day. *2 We measured the weight of the whole branch, including leaves and twigs. (TIF) [file pone.0256548.s002.tif]

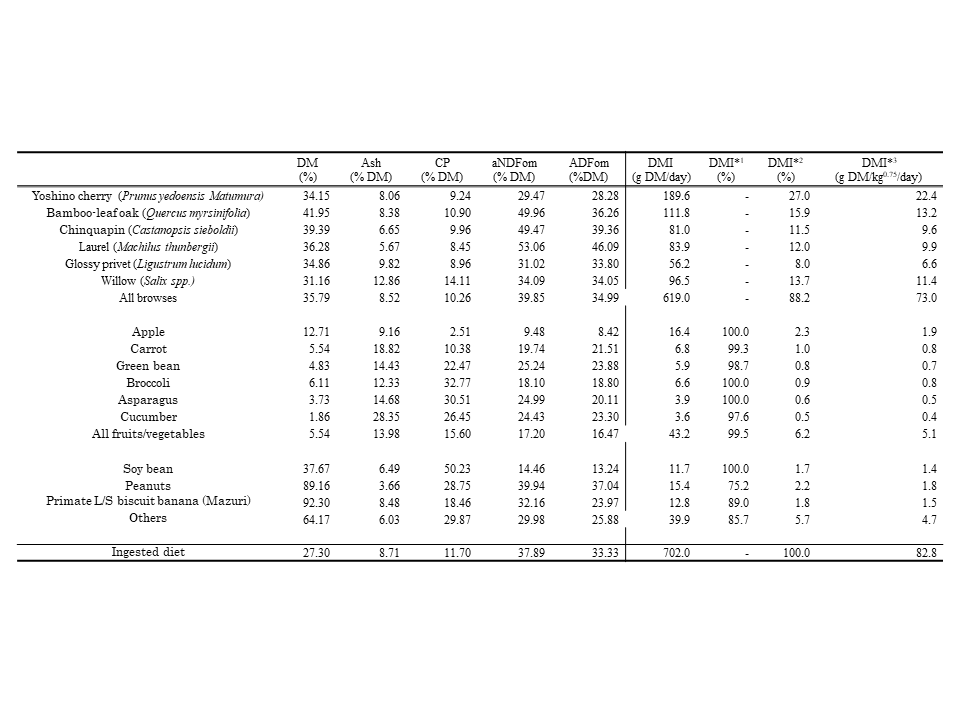

Supplement: S3 Table — DM, dry matter; ash, crude ash; CP, crude protein; aNDFom, neutral detergent fibre assayed with a heat stable amylase exclude residual ash; ADFom, acid detergent fibre excluded residual ash; DMI, dry matter intake; BW, body mass (kg). *1 Intake/offered of each feed (DM-basis). Browses were weighed as the whole branch, including leaves and twigs, but nutrient contents were analysed only for leaves. Thus, we could not calculate offered DM amounts of browse. *2 DM intake of each feed/total DM intake. *3 BW of the proboscis monkey was 17.3 kg at the beginning of the second week of the experiment. (TIF) [file pone.0256548.s003.tif]

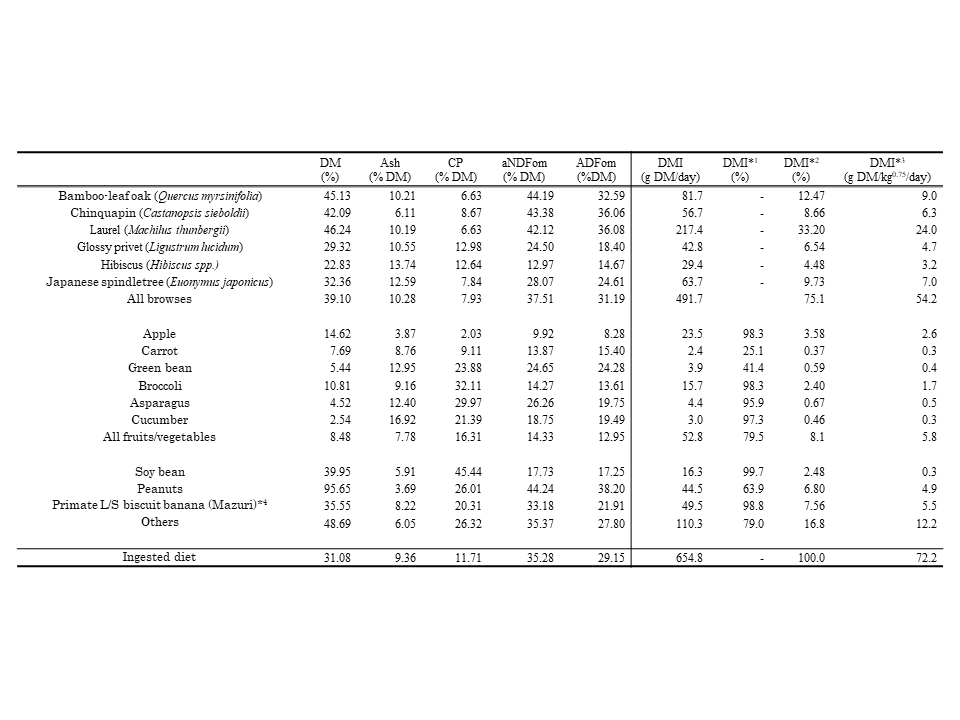

Supplement: S4 Table — DM, dry matter; ash, crude ash; CP, crude protein; aNDFom, neutral detergent fibre assayed with a heat stable amylase exclude residual ash; ADFom, acid detergent fibre excluded residual ash; DMI, dry matter intake; BW, body mass (kg). *1 Intake/offered of each feed (DM-basis). Browses were weighed as the whole branch, including leaves and twigs, but nutrient contents were analysed only for leaves. Thus, we could not calculate offered DM amounts of browse. *2 DM intake of each feed/total DM intake. *3 BW of the proboscis monkey was 18.9 kg.at the beginning of the second week of the experiment. *4 The pelleted feed was soaked in water before feeding. (TIF) [file pone.0256548.s004.tif]

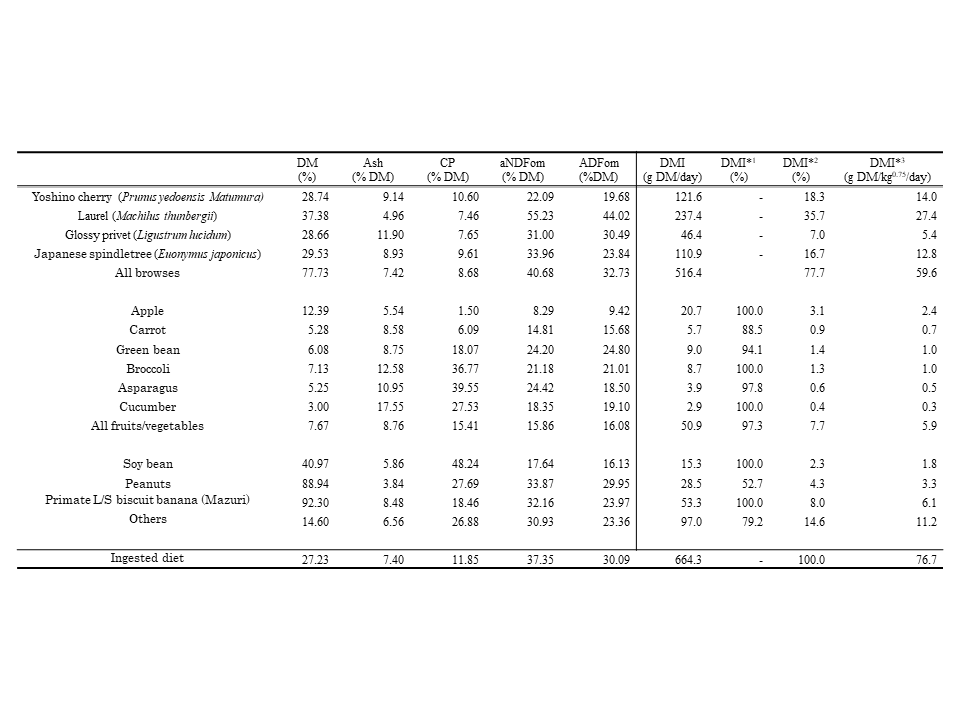

Supplement: S5 Table — DM, dry matter; ash, crude ash; CP, crude protein; aNDFom, neutral detergent fibre assayed with a heat stable amylase exclude residual ash; ADFom, acid detergent fibre excluded residual ash; DMI, dry matter intake; BW, body mass (kg). *1 Intake/offered of each feed (DM-basis). Browses were weighed as the whole branch, including leaves and twigs, but nutrient contents were analysed only for leaves. Thus, we could not calculate offered DM amounts of browse. *2 DM intake of each feed/total DM intake. *3 BW of the proboscis monkey was 17.8 kg at the beginning of the second week of the experiment. (TIF) [file pone.0256548.s005.tif]

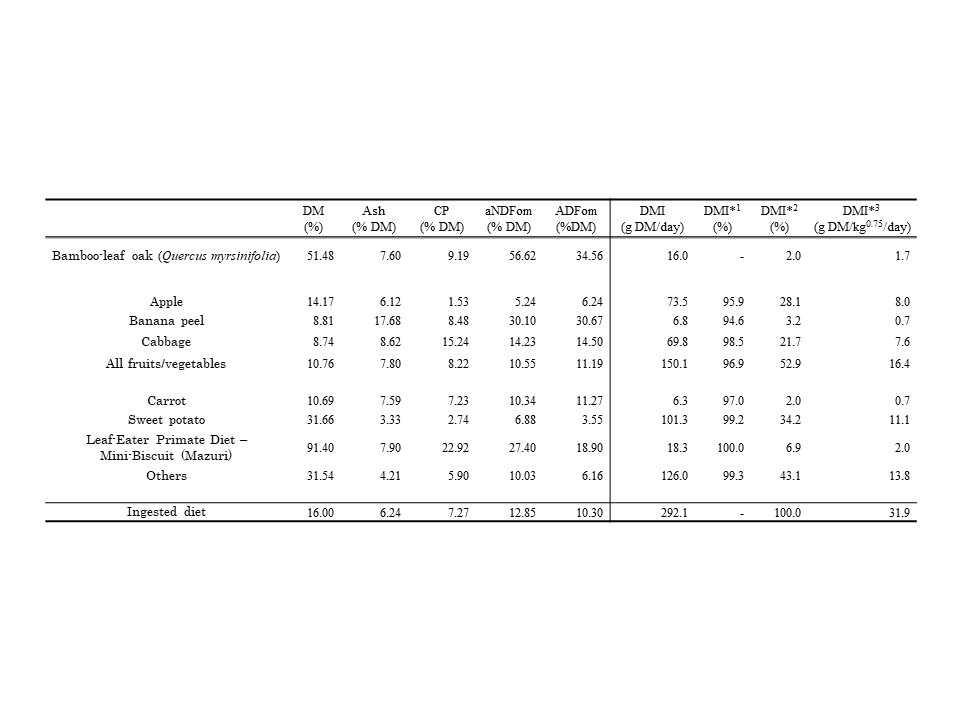

Supplement: S6 Table — DM, dry matter; ash, crude ash; CP, crude protein; aNDFom, neutral detergent fibre assayed with a heat stable amylase exclude residual ash; ADFom, acid detergent fibre excluded residual ash; DMI, dry matter intake; BW, body mass (kg). *1 Intake/offered of each feed (DM-basis). Browses were weighed as the whole branch, including leaves and twigs, but nutrient contents were analysed only for leaves. Thus, we could not calculate offered DM amounts of browse. *2 DM intake of each feed/total DM intake. *3 BW of two silver lutungs were 7.6 and 7.6 kg at the beginning of the second week of the experiment. (TIF) [file pone.0256548.s006.tif]

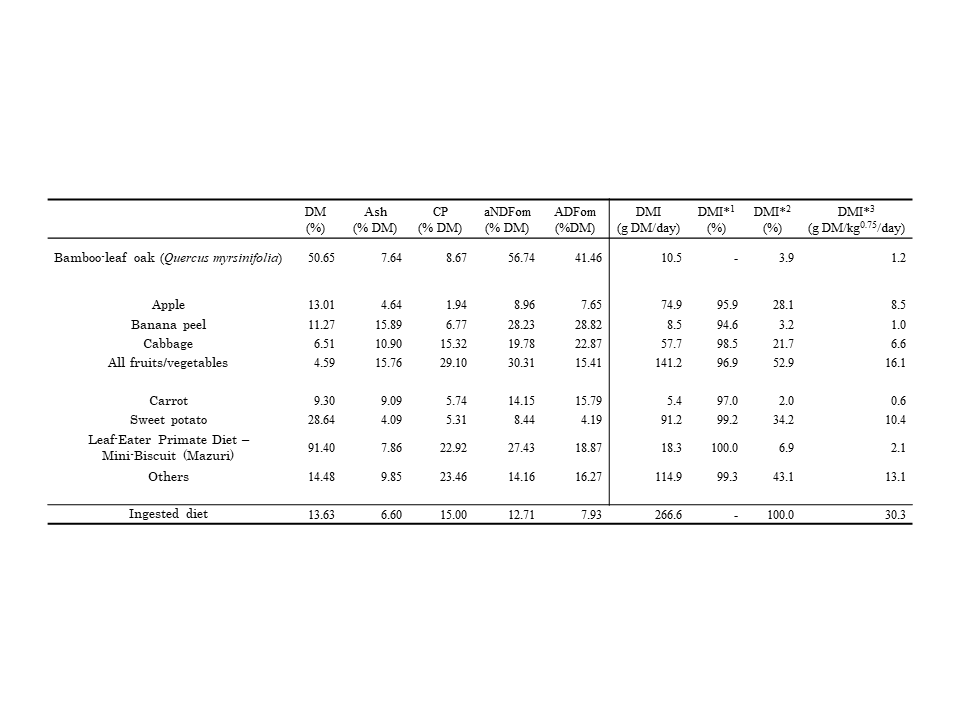

Supplement: S7 Table — DM, dry matter; ash, crude ash; CP, crude protein; aNDFom, neutral detergent fibre assayed with a heat stable amylase exclude residual ash; ADFom, acid detergent fibre excluded residual ash; DMI, dry matter intake; BW, body mass (kg). *1 Intake/offered of each feed (DM-basis). Browses were weighed as the whole branch, including leaves and twigs, but nutrient contents were analysed only for leaves. Thus, we could not calculate offered DM amounts of browse. *2 DM intake of each feed/total DM intake. *3 BW of two silver lutungs were 6.8 and 7.7 kg at the beginning of the second week of the experiment. (TIF) [file pone.0256548.s007.tif]
